# Emerging Roles of Calcium Signaling in the Development of Non-Alcoholic Fatty Liver Disease

**DOI:** 10.3390/ijms23010256

**Published:** 2021-12-27

**Authors:** Chien-Chih Chen, Li-Wen Hsu, Kuang-Den Chen, King-Wah Chiu, Chao-Long Chen, Kuang-Tzu Huang

**Affiliations:** 1 Department of Psychiatry, Kaohsiung Chang Gung Memorial Hospital, Kaohsiung 83301, Taiwan; chenfather@adm.cgmh.org.tw; 2 Liver Transplantation Center, Department of Surgery, Kaohsiung Chang Gung Memorial Hospital, Kaohsiung 83301, Taiwan; hsuliwen1230@gmail.com (L.-W.H.); dennis8857@gmail.com (K.-D.C.); c471026@ms6.hinet.net (K.-W.C.); clchen@cgmh.org.tw (C.-L.C.); 3 Institute for Translational Research in Biomedicine, Kaohsiung Chang Gung Memorial Hospital, Kaohsiung 83301, Taiwan; 4 Division of Hepato-Gastroenterology, Department of Internal Medicine, Kaohsiung Chang Gung Memorial Hospital, Kaohsiung 83301, Taiwan

**Keywords:** non-alcoholic fatty liver disease, calcium, endoplasmic reticulum, mitochondria, lysosome, autophagy, signal transduction

## Abstract

The liver plays a central role in energy metabolism. Dysregulated hepatic lipid metabolism is a major cause of non-alcoholic fatty liver disease (NAFLD), a chronic liver disorder closely linked to obesity and insulin resistance. NAFLD is rapidly emerging as a global health problem with currently no approved therapy. While early stages of NAFLD are often considered benign, the disease can progress to an advanced stage that involves chronic inflammation, with increased risk for developing end-stage disease including fibrosis and liver cancer. Hence, there is an urgent need to identify potential pharmacological targets. Ca^2+^ is an essential signaling molecule involved in a myriad of cellular processes. Intracellular Ca^2+^ is intricately compartmentalized, and the Ca^2+^ flow is tightly controlled by a network of Ca^2+^ transport and buffering proteins. Impaired Ca^2+^ signaling is strongly associated with endoplasmic reticulum stress, mitochondrial dysfunction and autophagic defects, all of which are etiological factors of NAFLD. In this review, we describe the recent advances that underscore the critical role of dysregulated Ca^2+^ homeostasis in lipid metabolic abnormalities and discuss the feasibility of targeting Ca^2+^ signaling as a potential therapeutic approach.

## 1. Introduction

The liver is a major site for lipid processing and storage. As such, liver dysfunction can lead to progressive lipid accumulation, a condition often predisposed to more severe diseases. Among the various hepatic lipid metabolic disorders, non-alcoholic fatty liver disease (NAFLD) is now the most common chronic liver disease worldwide, affecting approximately 25% of the population [1]. First described in the 1980s, NAFLD comprises a histological spectrum ranging from excess lipid deposition (hepatic steatosis) to the more aggressive form with consistent liver injury and inflammation (non-alcoholic steatohepatitis, NASH) and advanced fibrosis [2]. The presence of metabolic syndrome is a major risk factor for hepatic steatosis and NASH. Meanwhile, this association can be bidirectional, as NAFLD is becoming a risk factor for the comorbidities of metabolic syndrome, namely type 2 diabetes and hypertension [3]. Although there has been tremendous progress in the characterization of the pathogenesis of NAFLD, there are still significant unmet challenges in identifying effective therapeutic interventions, especially for advanced conditions.

Mechanistically, NAFLD represents an imbalance of metabolic activities that regulate the lipid uptake, synthesis, export and degradation. Free fatty acids are central to the pathogenesis of NAFLD. The major supply of lipids delivered to the liver comes in the form of free fatty acids following lipolysis of triglycerides in the adipose tissue. This action is highly regulated by insulin [4]. The liver is also capable of generating fatty acids through de novo lipogenesis (DNL), a process by which carbohydrates, especially fructose, are converted to fatty acids [5]. Free fatty acids are catabolized through β-oxidation to produce energy or esterified to form triglycerides. Triglycerides can be stored in lipid droplets or exported to the circulation as lipoproteins. When the hepatocytes are overwhelmed by excess fatty acids, lipotoxic fatty acid derivatives can form and contribute to ER stress, inflammasome activation and cell death, which are hallmarks of NAFLD progression [6]. Therefore, a better understanding of the natural history of NAFLD is essential for exploiting potential therapeutic strategies.

Calcium (Ca^2+^) is the most abundant mineral in the human body, primarily found in bones and teeth (in the form of hydroxyapatite). It plays a key role in maintaining the bone mass to support the skeletal system. The body is also constantly using Ca^2+^ in a wide range of biological activities, including muscle contraction and nerve impulse transmission [7]. At the cellular level, Ca^2+^ is a key signaling molecule involved in virtually all the important functions of cells, such as cell growth, differentiation, motility, metabolism and gene expression. Given the significance, the cellular concentration of Ca^2+^ must be tightly regulated. In resting cells, the cytoplasmic Ca^2+^ concentration is kept at low levels of about 10–100 nM. A high concentration of Ca^2+^ can lead to cellular damage; prolonged disruption of Ca^2+^ homeostasis may not trigger immediate cell death but often contributes to numerous chronic pathological developments [8]. Cellular Ca^2+^ homeostasis is maintained through a coordinated process that includes the participation of Ca^2+^ transporters, sensors and buffering proteins distributed throughout different compartments of the cell, such as the plasma membrane, cytoplasm, nucleus, mitochondria and the reticular network. In the context of metabolic regulation, for example, Ca^2+^ is essential for the secretion of insulin and glucagon from the pancreas. These hormones, in turn, control the metabolic responses of target tissues, such as gluconeogenesis, glycogen breakdown, lipid biosynthesis and ATP production, also in a Ca^2+^-dependent manner [9]. Aberrant Ca^2+^ signaling has been suggested to be one of the contributing factors in the development of hepatic steatosis. In this article, we will provide an overview of the mechanisms by which Ca^2+^ signaling modulates hepatic lipid metabolism and their involvement in the processes related to NAFLD development.

## 2. Alterations in Ca^2+^ Homeostasis and Organelle Dysfunction in Development of NAFLD

### 2.1. Basic Machinery of Ca^2+^ Signaling

Cytoplasmic Ca^2+^ is constantly maintained at a low level against a huge concentration gradient in the extracellular space and intracellular stores. This regulation is achieved by a highly integrated machinery that controls the spatiotemporal fluxes of Ca^2+^ into or out of the cell or intracellular stores, such as the endoplasmic reticulum (ER) and mitochondria. In response to hormones (e.g., glucagon, vasopressin and catecholamines), intracellular second messengers transmit the signals and regulate specific membrane receptors, called Ca^2+^ channels, to facilitate Ca^2+^ release from internal stores to the cytoplasm. One major second messenger is inositol-1,4,5-trisphosphate (InsP3), which triggers Ca^2+^ release from the ER via the InsP3 receptors (InsP3Rs) [10,11]. Another example of ER-located Ca^2+^ channel is the ryanodine receptor (RyR), whose activation is driven by an NAD^+^ derivative, cyclic ADP-ribose (cADPR), in excitable cells such as myocytes and neurons [12]. Additionally, nicotinic acid-ADP (NAADP) is crucial for Ca^2+^ release from lysosome-related acid compartments via two-pore channels (TPCs) [13].

Cytoplasmic Ca^2+^ signals normally come in the form of periodic oscillations from the opening of Ca^2+^ channels following hormone stimulation. The pattern of signals are usually repetitive spikes, with intervals ranging from milliseconds to minutes, depending on the type and concentration of the Ca^2+^-mobilizing agonists [14]. The rise of cytoplasmic Ca^2+^ is detected by the Ca^2+^ sensors, which are thought to play a prominent role in decoding Ca^2+^ oscillations. These Ca^2+^ sensors, upon Ca^2+^ binding, undergo conformational changes enabling them to interact with downstream targets and transduce Ca^2+^ signals. One of the best known Ca^2+^ sensors is calmodulin (CaM), which modulates the activity of an array of target proteins such as Ca^2+^/CaM-dependent protein kinase (CAMK) and calcineurin and myosin light chain kinase (MLCK) [15]. Other notable Ca^2+^ sensors include troponin and the neuronal Ca^2+^ sensor (NCS) family proteins, which are crucial for muscle contraction and neurotransmission, respectively [16,17].

After a hormonal activation event, excess Ca^2+^ is removed from the cytoplasm via the action of various Ca^2+^ pumps to effectively maintain Ca^2+^ at very low concentration levels. These Ca^2+^ pumps, which include the sarco/endoplasmic reticulum calcium ATPases (SERCAs), plasma membrane Ca^2+^-ATPase (PMCA) and secretory pathway calcium ATPase (SPCA), use the energy of ATP to actively transport Ca^2+^ to the ER, extracellular space and Golgi complex, respectively [18]. In the case of large cytoplasmic Ca^2+^ variations, the calcium/sodium exchanger (NCX) at the plasma membrane and mitochondrial Ca^2+^ uniporter (MCU) can support Ca^2+^ extrusion due to their high capacity for Ca^2+^ transport [19,20]. The concerted action of these mechanisms helps reestablish the resting cellular Ca^2+^ levels. The ER has a high capacity for Ca^2+^ storage and release (in the high micromolar range) due to the abundant expression of Ca^2+^-buffering proteins, such as calreticulin, which accounts for nearly half of the total ER Ca^2+^ binding [21]. Several other Ca^2+^-binding proteins, such as GRP94 (endoplasmin), calnexin and BiP/GRP78, also participate in ER Ca^2+^ buffering. Many of the ER Ca^2+^-buffering proteins also serve as ER chaperones, important in protein folding and processing.

### 2.2. Disruption of Ca^2+^ Homeostasis in the ER in NAFLD

**Figure 1:**
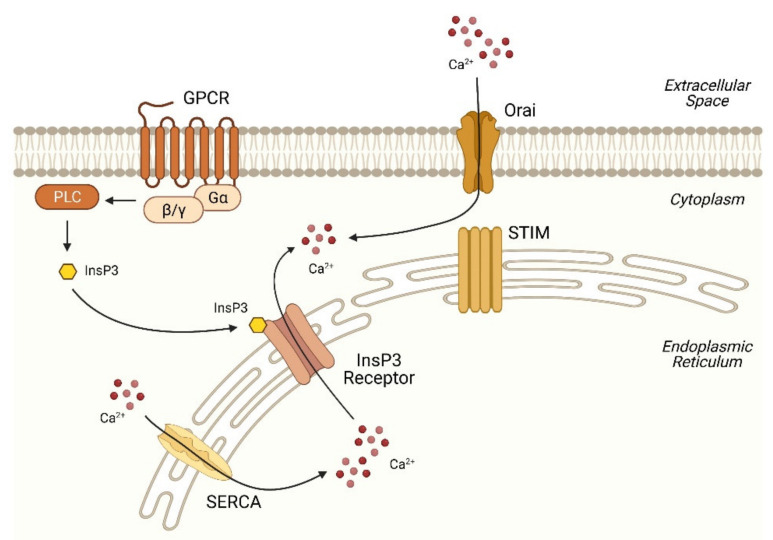
Schematic representation of Ca^2+^ mobilization in the ER. Upon activation of the cell surface GPCRs, PLC action results in the formation of InsP3. The binding of InsP3 to the InsP3 receptor triggers Ca^2+^ release from the ER to the cytoplasm. Following Ca^2+^ release, the dissociation of Ca^2+^ from the STIM proteins leads to STIM oligomerization and interaction with the plasma membrane Ca^2+^ channel Orai, allowing Ca^2+^ to enter the cell from the extracellular space. Conversely, cytoplasmic Ca^2+^ is withdrawn to the ER via SERCA activity. Abbreviations: GPCR, G-protein-coupled receptor; PLC, phospholipase C; InsP3, inositol-1,4,5-trisphosphate; STIM, stromal interaction molecules; SERCA, sarco/endoplasmic reticulum calcium ATPase.

The ER represents a major Ca^2+^ store. Ca^2+^ mobilization is most commonly initiated by activation of the G-protein-coupled receptors (GPCRs) (Figure 1). Upon receptor activation, the associated heterotrimeric G-protein (which has three subunits: α, β and γ) is activated. GPCRs are classified based on the type of Gα subunit activated, with each type having its own signaling mechanisms. For example, glucagon binds the glucagon receptor and activates the Gαs protein, resulting in the stimulation of adenylate cyclase and production of cyclic adenosine monophosphate (cAMP). Increased cAMP further leads to the activation of protein kinase A (PKA), which is able to phosphorylate InsP3R, triggering Ca^2+^ release from the ER [22,23]. Alternatively, glucagon also activates Gαq and then induces hydrolysis of the membrane lipid phosphatidylinositol 4,5-bisphosphate (PIP2) via the enzyme phospholipase C (PLC), leading to the formation of InsP3 and diacylglycerol (DAG) [24]. The binding of InsP3 to the Ca^2+^ channel InsP3R on the surface of ER, as described in the previous section, triggers channel opening and Ca^2+^ release into the cytoplasm. DAG, a classic protein kinase C (PKC) activator, can further modulate Ca^2+^ signaling [25,26]. In addition to glucagon, whose contribution in hepatic lipid metabolism has been well-recognized [27], various other GPCRs also play a key role in NAFLD development. Activation of the β-adrenergic receptors (receptors for catecholamines such as epinephrine and norepinephrine) is associated with increased hepatic lipid accumulation during aging [28]. Bile acids can signal through sphingosine-1-phosphate receptor 2 and regulate hepatic lipid metabolic genes [29]. Moreover, free fatty acid receptors (FFARs), previously identified as orphan Gq-coupled receptors, mediate hepatic metabolic signaling in response to free fatty acids and have become new therapeutic targets for NAFLD [30].

The Ca^2+^ release results in the depletion of Ca^2+^ in the ER. The stromal interaction molecules STIM1 and STIM2 are ER resident Ca^2+^ sensors that can detect the decrease of ER Ca^2+^ through their luminal N-terminal Ca^2+^-binding domains (termed EF-hand domains) [31]. The EF-hand domain, identified in several hundreds of cellular proteins, is a well-conserved helix–loop–helix structure with high Ca^2+^-binding affinity, allowing EF-hand proteins to sense the changes in the intracellular Ca^2+^ concentration [32]. Upon ER Ca^2+^ depletion, STIMs undergo rapid oligomerization and translocation towards the junction of ER and the plasma membrane, enabling recruitment and activation of the plasma membrane Ca^2+^ channel proteins Orai, which form the Ca^2+^ release-activated Ca^2+^ (CRAC) channels [33]. Ca^2+^ influx from the extracellular space through Orai channels leads to increased cytoplasmic Ca^2+^ levels and downstream signaling, a process known as store-operated Ca^2+^ entry (SOCE). Excess Ca^2+^ is then pumped back to the ER via SERCAs to replenish the deficit, maintaining a dynamic Ca^2+^ balance.

Recent studies have implicated the mishandling of Ca^2+^ by the ER as the basis of metabolic dysfunction in NAFLD. Abnormal regulation of Ca^2+^ transport proteins are observed in the steatotic liver. One study showed a moderate increase in Orai1 expression in high fat diet-induced NAFLD and fatty acid-treated hepatocytes [34]. In another study using palmitate-treated and obese Zucker rat hepatocytes, SOCE was substantially inhibited via a PKC-dependent mechanism, while the expression of STIM and Orai isoforms remained unchanged [35]. The inhibition is associated with a decreased ER luminal Ca^2+^ concentration and decreased expression/activity of SERCA2b ATPase, the major isoform of SERCA protein in the liver [36,37]. In the obese liver, de novo lipogenesis and phospholipid synthesis genes are stimulated, resulting in lipid droplet accumulation and an increase in the phosphatidylcholine (PC)-to-phosphatidylethanolamine (PE) ratio. The imbalance of the PC/PE ratio changes the ER membrane fluidity and impairs SERCA2b activity, thereby contributing to protein misfolding and ER stress, both of which are indications of NAFLD progression [36]. It has also been shown that the PKC-dependent SERCA inhibition by palmitate treatment in hepatocytes causes autophagy arrest and lipotoxicity [38,39]. These results strengthen the critical role of SERCA dysfunction in NAFLD development. Indeed, obesity-induced hepatic steatosis and ER stress can be alleviated by the overexpression of SERCA2b in mice [37].

Another crucial player in the maintenance of hepatocyte Ca^2+^ homeostasis is the InsP3-gated Ca^2+^ release channel InsP3R. The implication of InsP3R in lipid metabolic regulation was established in a recent report that InsP3R *Drosophila* mutants exhibited excess body weight and fat deposits even on a normal diet [40]. In mammalian species, the InsP3R family is composed of three closely related isoforms (about 70% homology), each encoded by a distinct gene transcript. These isoforms are among some of the largest ion channels in the cell (~2700 amino acids) and further assemble into homo- or hetero tetramers of ~1200 kDa [41,42]. The InsP3R isoforms differ in their expression among tissues and have a different affinity for InsP3 [43]. In the liver, InsP3R1 and InsP3R2 are the predominant isoforms, with distinct patterns of subcellular localization [44]. InsP3R3 is physiologically at undetectable to low levels, and its overexpression is associated with hepatocellular carcinogenesis [45].

In terms of hepatic lipid regulation, an increased expression of InsP3R1 is observed in patients with NASH [46]. This is in concert with observations in hepatocyte cellular models in which InsP3R1 was upregulated upon palmitate treatment. The upregulation is due to enhanced protein stability through the Src-dependent tyrosine phosphorylation of InsP3R1 [47]. However, the InsP3R1 levels remained unaltered in a high-fat diet and ob/ob mouse models, suggesting differences in the regulatory mechanisms between species [48]. It was later discovered that it is not only the overall expression but also subcellular localization of InsP3R1 that is important in the maintenance of metabolic homeostasis. Arruda et al. demonstrated that, in the liver, obesity drives a marked increase in InsP3R1 localization at the ER–mitochondria contact sites, defined as the mitochondria-associated ER membranes (MAMs), thereby resulting in an overload of Ca^2+^ flux to the mitochondria and production of reactive oxygen species (ROS) [49]. Increased MAMs are also shown to be correlated with the disease severity of NAFLD in human liver specimens [46]. This concept was, however, challenged by another study using in situ proximity ligation assays demonstrating that, contrary to enrichment, disruption of the MAM integrity is associated with insulin resistance and fatty liver formation [50]. These discrepancies may be due to examination at different stages of the disease, among other potential regulatory events. Further delineation will be much required.

Moreover, liver-specific InsP3R1 knockout mice exhibited reduced hepatic triglyceride accumulation and were resistant to high fat diet-induced fatty liver [46], further strengthening the crucial role of InsP3R1 in the development of NAFLD. On the other hand, hepatic InsP3R2 was downregulated in both rats on a high-fat, high-fructose diet and patients with NAFLD and NASH. However, InsP3R2 knockout mice have no apparent lipid metabolic phenotype. It was further learned that a decreased expression of InsP3R2 may account for the impaired liver regeneration that often occurs in NAFLD patients [51]. These findings suggest a critical role of ER Ca^2+^ homeostasis in maintaining metabolic functions.

### 2.3. Disturbances in Mitochondrial Ca^2+^ Homeostasis in NAFLD

**Figure 2:**
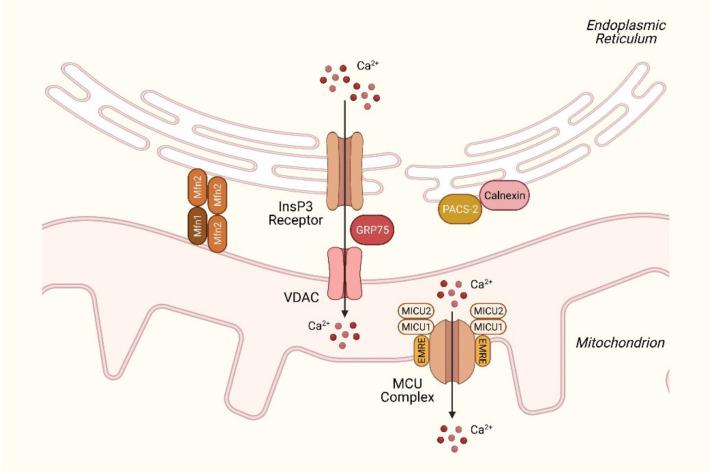
Schematic representation of Ca^2+^ flux at the ER–mitochondria contact sites. The ER and mitochondria are functionally coupled through physical interactions at contact sites (also known as MAMs), allowing Ca^2+^ transport from the ER to mitochondria through the InsP3 receptor and VDAC. The two ion channels are connected by the chaperone GRP75. The MAM structure is stabilized by tethering proteins Mfn1/2 and interactions between calnexin and PACS-2. Ca^2+^ enters the mitochondrial matrix via the MCU complex at the inner mitochondrial membrane. MCU-binding proteins MICU1/2 control the opening of the channel, while EMRE assists the MCU–MICU interaction and regulates the complex activity. Abbreviations: InsP3, inositol-1,4,5-trisphosphate; Mfn1/2, mitofusin 1/2; VDAC, voltage-dependent anion channel; MCU, mitochondrial Ca^2+^ uniporter; MICU1/2, mitochondrial Ca^2+^ uptake 1/2; EMRE, essential MCU regulator.

The ER–mitochondria contacts (MAMs) have been shown to be an essential character of metabolic disturbances. Functionally, MAMs play a pivotal role in Ca^2+^ signaling and the associated cellular functions, including bioenergetics, lipid trafficking and cell death [52]. Under physiological conditions, the mitochondrial Ca^2+^ concentration is at comparable levels (~100 nM) to that in the cytoplasm. The close proximity between the ER and mitochondria creates Ca^2+^ microdomains (with concentrations up to 10–50 μM), allowing the rapid transport of Ca^2+^ to the mitochondria when needed [9]. Ca^2+^ uptake is mediated by the voltage-dependent anion channel (VDAC) of the outer mitochondrial membrane, which interacts with the InsP3R1 on the ER through linkage with molecular chaperone glucose-regulated protein 75 (GRP75) [53] (Figure 2). This type of transport is driven by the electrochemical gradient across the mitochondrial membrane, regulating the entry of Ca^2+^ and other metabolites such as pyruvate, succinate and NADH [54]. The knockdown of GRP75 abolishes the functional coupling of MAMs and decreases Ca^2+^ transfer to the mitochondria [53].

The isolation of subcellular fractions has helped identify numerous MAM resident proteins, including those that regulate MAM integrity and Ca^2+^ permeability [55]. Mitofusin 2 (Mfn2), best known for its role in mitochondrial fusion, is crucial for tethering ER to the mitochondria and the maintenance of MAM stability by establishing homo- or heterotypic (with Mfn1) interactions. The ablation of Mfn2 is also reported to disrupt ER–mitochondria interactions and reduces Ca^2+^ uptake [56]. However, the role of Mfn2 at ER–mitochondria contacts remains debated, as research groups deliver conflicting results regarding Mfn2′s tethering function by using various imaging techniques and biochemical analyses [57,58]. In terms of the effects on the lipid metabolism, a decreased expression of Mfn2 was found in NAFLD mouse models (high-fat diet and methionine-choline-deficient) and NASH patients. The hepatic ablation of Mfn2 also results in impaired insulin signaling and increased ER stress. This is, at least in part, due to defects in phospholipid synthesis and reduced phosphatidylserine transfer from the ER to the mitochondria, leading to ER stress and the NASH phenotype [59,60]. Indeed, as described in the previous section, aberrant phospholipid composition in the ER membrane is a potent initiator of ER stress and can thus lead to NAFLD progression [36]. Proteins that are involved in phospholipid synthesis and trafficking, such as phosphatidylethanolamine N-methyltransferase (PEMT), may be promising targets for research [61].

Ca^2+^ transport towards the mitochondrial matrix occurs through the MCU located in the inner mitochondrial membrane [62] (Figure 2). The MCU is regulated by a number of interacting proteins, including mitochondrial Ca^2+^ uptake 1/2 (MICU1/2), essential MCU regulator (EMRE) and MCU regulator 1 (MCUR1), creating a large pore-forming complex. To prevent Ca^2+^ overload, Ca^2+^ influx is counterbalanced by an extrusion mechanism mediated by the mitochondrial Na^+^/Ca^2+^ exchanger NCLX [63]. Under resting conditions, MCU activity is inhibited by MICU2. The increase in Ca^2+^ concentration induces a conformational change, which abolishes MICU2 inhibition and, in turn, activates the MCU, while MICU1 acts as a coactivator and allows Ca^2+^ to enter the mitochondrial matrix [64]. EMRE is responsible for the interaction between MCU and MICU1/2 and is essential for MCU-mediated Ca^2+^ transport [65]. MCUR1 functions as a scaffolding factor of the MCU complex and is involved in Ca^2+^-dependent mitochondrial metabolism [66].

The MCU complex finely adjusts mitochondrial Ca^2+^ bioenergetics according to cell demands and contributes to the regulation of various hepatic functions, including lipid and carbohydrate metabolism, proliferation and apoptosis. Physiologically, increased Ca^2+^ stimulates aerobic metabolism, NADH formation and, consequently, respiratory chain activity, enhancing ATP production. Four mitochondrial dehydrogenase enzymes have been described to be Ca^2+^-dependent. Glycerol-3-phosphate dehydrogenase (GPDH) is located in the inner mitochondrial membrane and essential for the glycerol phosphate shuttle in oxidative phosphorylation. The other three are matrix enzymes—pyruvate dehydrogenase phosphatase, which modulates pyruvate dehydrogenase activity, isocitrate dehydrogenase (IDH) and oxoglutarate dehydrogenase (OGDH), major enzymes in the citric acid cycle [67]. The Ca^2+^-mediated activation of citric acid cycle dehydrogenases results in elevated NADH levels, increasing the mitochondrial energy metabolism and ATP synthesis. In fact, Ca^2+^ also directly modulates the activity of the election transport chain complexes and ATP synthase [68]. On the other hand, excess Ca^2+^ accumulation has deleterious effects. Ca^2+^ overload can lead to the overproduction of ROS by the respiratory chain and opening of the mitochondrial permeability transition pore (mPTP). This triggers depolarization of the inner mitochondrial membrane and collapse of the membrane potential, with a consequent release of cytochrome c to the cytoplasm and induction of a cascade of apoptotic events [69].

Given the pivotal role of Ca^2+^ dependence in mitochondrial oxidative metabolism, loss-of-function animal models have been generated and studied by several research groups. Whole-body MCU knockout is embryonically lethal in C57BL/6 mice and, however unexpectedly, viable in mice on a CD-1 background [70]. The conditional knockout of MCU in the liver results in Ca^2+^ depletion in the mitochondrial matrix and impaired oxidative phosphorylation. More importantly, a lack of mitochondrial Ca^2+^ sequestration leads to delayed cytoplasmic Ca^2+^ clearance and promotes lipid accumulation due to dephosphorylation of the AMP-activated protein kinase (AMPK) [71]. Moreover, MICU1 knockout mice display a failure of basic vital functions and die a few hours after birth. The liver-specific knockdown of MICU1 demonstrates severe impairment in tissue regeneration after partial hepatectomy due to unresolved inflammation and cell cycle defects [72]. These results highlight the importance of MCU-mediated Ca^2+^ handling as a crucial mechanism in controlling hepatic metabolism.

### 2.4. Dysregulation in Lysosomal Ca^2+^ Signaling in NAFLD

The endolysosomal system is widely known for its role in membrane trafficking and macromolecule degradation. It is also an essential cellular compartment in which signals from hormones, nutrients and growth factors converge to coordinate various responses [73]. Endolysosomal Ca^2+^ has recently emerged as one of the major players in modulating intracellular Ca^2+^ signaling although numerous unknowns remain to be elucidated.

The lysosome is a significant intracellular Ca^2+^ store with an estimated capacity of ~0.5 mM. The vacuolar (V)-type H^+^-ATPase establishes an H^+^ gradient across the lysosomal membrane and provides the energy required for Ca^2+^ entry through Ca^2+^/H^+^ exchangers [74]. The release of lysosomal Ca^2+^ can be triggered by several Ca^2+^-mobilizing messengers, including NAADP and phosphatidylinositol 3,5-bisphosphate [PI(3,5)P2], via Ca^2+^-permeable channels, e.g., TPCs and transient receptor potential mucolipins (TRPMLs).

TRPMLs are evolutionarily conserved, nonselective cation channels containing six membrane-spanning domains and function in tetrameric forms. In mammals, the TRPML channel family has three members, with each member having partially overlapping biological properties but different tissue distribution and subcellular localization. TRPML1 is expressed ubiquitously in all tissues, whereas TRPML2 and TRPML3 show more functional specificity in various cell types [75].

TPCs are members of the voltage-gated ion channel superfamily. Structurally, TPCs function as homodimers, with each subunit containing two six-transmembrane domain repeats connected by a linker region [76]. The TPC family has three members, although TPC3 is not detected in humans and rodents [77]. TPCs were originally postulated to be the targets for the Ca^2+^ mobilizing messenger NAADP [13]. However, the molecular mechanisms of how NAADP activates these channels and whether directly or indirectly have been a matter of many discussions over the past years. Two research groups have further reported that TPCs are instead Na^+^ release channels that can be activated by [PI(3,5)P2] and by voltage [78,79]. Despite these discrepancies, a recent study using small molecule agonists of TPC2 has provided new insight into the potential mechanism by which the coregulation of TPCs by these disparate stimuli can occur with specific permeation properties [80]. In addition, as binding sites for NAADP have not been characterized on NAADP-sensitive ion channels, there have been hypotheses that NAADP action may be mediated by unidentified NAADP-binding protein(s) that contribute to NAADP sensitivity. Two independent research works have recently demonstrated that a 23-kDa protein, identified as Jupiter microtubule-associated homolog 2 (JPT2), associates with NAADP and, in turn, facilitates Ca^2+^ release [81,82].

One of the major functions of the endolysosomal system is membrane trafficking through the interconnected vesicular network, acting as a sorting station to process internalized cargo. In the liver, cholesterol-enriched low-density lipoprotein (LDL) is taken up by the LDL receptor on the plasma membrane and internalized through endocytosis. The LDL-bound LDL receptor is then sorted in the endosome: the receptor proteins are transported back to the plasma membrane, while LDL cholesterol that exits from the late endosomes, and lysosomes is either processed to bile acids or to cholesterol esters for storage [83]. In a report by Grimm et al., TPC2-deficient cells had profound trafficking defects in the endolysosomal degradation pathway. More importantly, TPC2-deficient mice were more susceptible to NAFLD when fed a high-cholesterol diet. This is due to impaired LDL trafficking and is not associated with altered pH or lysosomal proteolytic activity [84]. These results are independent of the traditional risk factors for metabolic diseases and may provide a new area of research in studying susceptibility to NAFLD.

The lysosome not only acts as the cell’s recycling center but also mediates a tightly regulated adaptive response to energy-demanding conditions widely known as autophagy. Autophagy is a well-conserved degradative process through which cellular organelles and macromolecules are disassembled during times of nutrient deprivation and other threats to cellular homeostasis. Three types of autophagy have been categorized based on the mechanisms of cargo sequestration: macroautophagy, microautophagy and chaperone-mediated autophagy (CMA). The detailed mechanistic distinctions of these autophagic pathways have been discussed in several recent elegant reviews and will not be covered here [85,86].

Growing evidence indicates that lysosomal Ca^2+^ is a critical modulator of autophagy. It has been proposed that, during nutrient deprivation, Ca^2+^ is released from the lysosome through TRPML1, which creates high Ca^2+^ microdomains, leading to activation of the Ca^2+^-sensitive serine/threonine phosphatase calcineurin and dephosphorylation of transcription factor EB (TFEB) [87]. TFEB is considered the master regulator of lysosomal function and plays a key role in induction of the gene expression related to lysosomal biogenesis and autophagy [88]. In nutrient-rich conditions, TFEB is phosphorylated by the mTORC1 complex and sequestered in the cytoplasm through 14-3-3 protein binding. Dephosphorylated TFEB is dissociated with cytoplasmic 14-3-3 proteins and translocated to the nucleus, where it initiates lysosomal target gene expression [89]. Interestingly, TRPML1 can also induce autophagic vesicle formation through the activation of Ca^2+^/CaM-dependent protein kinase kinase β (CaMKKβ) and AMPK, an observation independent of TFEB [90], further suggesting lysosome as an important hub for autophagy and cell metabolism.

The process by which intracellular lipid droplets are catabolized by autophagy is also referred to as lipophagy [91]. Lipophagy is a subset of the so-called selective autophagy in which specific types of cell organelles are removed and recycled by the lysosomal pathway. Selective autophagy often relies on receptors or regulatory proteins as a way of recognition to ensure target specificity [92]. Ever since the discovery of lipophagy in hepatocytes over a decade ago, there have been numerous studies focused on elucidating its physiological role and implications in lipid metabolic disorders, including NAFLD [93]. While the contribution of Ca^2+^ signaling in canonical autophagy is more established, studies specifically investigating the role of Ca^2+^ regulation in lipophagy, especially in the context of liver disease, are relatively few. As discussed in Section 2.2, hepatic SOCE is impaired in obese or lipid-laden conditions. This is in parallel to the accumulation of p62/Sequestosome-1, an autophagosome cargo protein, which represents a blockade of autophagy flux [94]. Moreover, SOCE-deficient cells (from STIM1/2 inducible knockout mice or STIM1, ORAI1 loss-of-function mutations) have reduced lipolysis and impaired lipid droplet mobilization but, interestingly, increased lipophagy [95]. This is likely a compensatory mechanism that protects the cells from lipotoxicity, indicating an interconnective nature of the lipid catabolic pathways to maintain homeostasis.

### 2.5. Aberrant Ca^2+^ Signaling in Hepatic Nonparenchymal Cells in NAFLD

During the course of NAFLD progression, in addition to hepatocytes, which are the primary target of lipotoxicity and oxidative stress, hepatic nonparenchymal cells are also crucial contributors. The nonparenchymal cells represent about 20% of the total liver mass and are composed of Kupffer cells, hepatic stellate cells (HpSCs), liver sinusoidal endothelial cells and other cell types. These specialized cells interact with hepatocytes and play a key role in liver regeneration and inflammatory responses.

Liver-resident Kupffer cells are the major hepatic macrophages in healthy livers and essential components of the innate immunity. Kupffer cells are central in modulating local inflammation, tissue remodeling and metabolic processes [96]. Upon lipotoxic injury, Kupffer cells are activated and release chemokines to recruit circulating leukocytes, including monocytes, which further differentiate into distinct subsets of monocyte-derived macrophages. The diversity of hepatic macrophage subsets reflects different functional responses to the environmental signals [97]. The lysosome-associated mTORC1 has been shown to play a pivotal role in hepatic macrophage polarization. When exposed to excessive lipids, hepatic macrophages adopt the classical M1 phenotype, characterized by the increased production of proinflammatory cytokines such as tumor necrosis factor-α (TNF-α), interleukin-6 (IL-6) and IL-1β [98]. The activation of mTORC1 increases cytoplasmic Ca^2+^ levels, thereby promoting (V)-type H^+^-ATPase-dependent lysosome acidification and lysosomal lipolysis. This results in an increase in the alternative M2 macrophages, a protective response to prevent NAFLD progression [99].

The cytokines released by Kupffer cells and monocyte-derived macrophages activate HpSCs, the main effector cells that initiate fibrogenic events. Upon activation, HpSCs undergo morphological and functional changes, transdifferentiating into a myofibroblast-like phenotype. Activated HpSCs produce contractile cytoskeletal proteins and components of the extracellular matrix, which may further lead to cirrhotic changes associated with hepatic dysfunction in advanced disease [100]. The activation of HpSCs involves Ca^2+^ mobilization. In activated HpSCs, ER Ca^2+^ transport proteins InsP3R1, SERCA2b and Ca^2+^ buffers calnexin and calreticulin are upregulated [101]. Ca^2+^ transients are induced downstream of the cell surface GPCRs (e.g., vasopressin, P2Y and 5-hydroxytryptamine (5-HT) receptors), which mediate HpSC contractility, proliferation and gene expression [101,102,103]. The blockade of Ca^2+^ signaling abrogates nuclear calmodulin kinase II-mediated Cdc25 phosphorylation and, thus, results in cell cycle arrest [104]. In contrast, imbalance in Ca^2+^ homeostasis causes ER stress and, eventually, HpSC apoptosis. The accumulation of intracellular Ca^2+^ increases the phosphorylation of the c-Jun N-terminal kinase (JNK) and p38, followed by the activation of Ca^2+^-dependent cysteine proteases calpains and caspase-12/caspase-3 processing [105]. These results suggest a crucial role of Ca^2+^ in both the initiation and resolution of HpSC activation.

## 3. Ca^2+^ Signaling as a Therapeutic Target for NAFLD

Based on what we have discussed in the previous sections, a general idea of Ca^2+^ being a critical intracellular messenger for lipid metabolism can be acknowledged. Hepatic lipid accumulation leads to SERCA dysfunction, resulting in the elevation of cytoplasmic Ca^2+^ and ER stress. Therefore, treatment options that target Ca^2+^ signaling to maintain optimal Ca^2+^ homeostasis is a rational approach and can potentially be beneficial. Indeed, the overexpression of SERCA2b in obese mice alleviates ER stress and hepatic steatosis [37]. The treatment of obese mice with a small molecule of SERCA2 activator CDN1163 also improves the metabolic parameters and inhibits NAFLD progression [106]. As numerous Ca^2+^ channels/pumps are responsible for normal physiological functions, a challenge for drug development would be to ensure these compounds are specific to the relevant pathways and do not interfere with normal cellular function of the target tissue or even other off-target tissues. The overexpression of SERCA2b or CDN1163 treatment in normal lean mice does not impair energy homeostasis and alter the body fat composition [37,106], indicating that manipulating SERCA expression or activity has little effect when ER Ca^2+^ is within the normal physiological levels.

In addition to ER stress, lipotoxicity-induced cytoplasmic Ca^2+^ increase is also responsible for attenuation of the autophagy flux. Park et al. therefore proposed that the inhibition of cytoplasmic Ca^2+^ influx by using Ca^2+^ channel blockers may be beneficial for ameliorating the autophagy defects and the pathological consequences in obesity. They show that, in mice on a high-fat diet, the administration of verapamil, a non-dihydropyridine Ca^2+^ channel blocker commonly used in the treatment of high blood pressure and heart arrhythmias, can restore autophagy flux in the liver and reduce obesity-induced pathologies, including hepatic steatosis and insulin resistance [107]. Despite the positive results, it is nevertheless noteworthy that this has only been examined in a preclinical setting and has not been applied in human trials. In addition, not all Ca^2+^ channel blockers can achieve similar results. A number of those have proven to have no or even negative effects on glucose or lipid metabolism [108]. Further large-scale investigations are required to confirm these previous findings.

A number of pharmacological agents, although not specifically designed to target Ca^2+^ channels or transporters, have shown the ability to modulate Ca^2+^ signaling and potentially improve metabolic abnormalities. The glucagon-like peptide-1 (GLP-1) receptor agonists exendin-4 and exenatide can alleviate hepatic steatosis by correcting impaired SOCE in hepatocytes [109,110]. Liraglutide, another GLP-1 receptor agonist widely used for type 2 diabetes treatment, attenuates high-fat diet-induced NAFLD through a TFEB-dependent mechanism [111]. Maresin 1, a macrophage-derived chemical mediator for inflammation resolution, has been shown to suppress ER stress and hepatic steatosis in high-fat diet-fed mice via the activation of AMPK, thereby augmenting SERCA2b expression and relieving the autophagy blockade [112,113]. Jaceosidin, a natural flavonoid isolated from the herb *Artemisia vestita*, has also been reported to reduce hepatic lipid accumulation and ameliorate insulin resistance in obese mice through the upregulation of SERCA2b [114]. These studies highlight the therapeutic potential of Ca^2+^-signaling modulators—in particular, those that regulate SERCA activity in the treatment of NAFLD-associated metabolic pathologies.

## 4. Conclusions and Future Perspectives

Ca^2+^, as an intracellular second messenger, modulates a myriad of cellular processes. These actions involve an intricate set of regulatory proteins that allow interplay between the Ca^2+^ stores (ER, mitochondria and lysosome) and transmit coordinated Ca^2+^ signals. During the past decade, significant progress has been made towards the understanding of Ca^2+^ signaling in regulation of the hepatic lipid metabolism. In this review, we have summarized some of the crucial mechanisms among different cellular organelles and highlighted the association between disrupted Ca^2+^ homeostasis and NAFLD development (Table 1). These changes can further alter the systemic energy metabolism and impact the overall health. Recently, a group of international experts reached a consensus that the term NAFLD does not fully reflect the current emphasis on the crucial role of metabolic dysfunction in disease development and proposed a more appropriate term: metabolic (dysfunction)-associated fatty liver disease (MAFLD) [115]. From our perspective, impaired Ca^2+^ signaling is highly related to hepatic metabolic dysfunction and should be recognized as a critical element of this updated term. Developing therapies that target defective key factors and pathways, such as the SERCA enzyme and autophagy pathway, to correct concomitant Ca^2+^ dysregulation have become promising approaches to treat these metabolic disorders. However, many of the pharmacological agents are still at an early stage for therapeutic intervention and require further investigations.

In this review, we mainly discussed the involvement of Ca^2+^ channel/pumps and their auxiliary cofactors. However, Ca^2+^-buffering proteins/chaperones may also have critical roles in energy metabolism, although their implications in metabolic diseases are less-studied. For example, the ER chaperone calnexin is enriched at the MAM connections and stabilizes the MAM structure by binding to phosphofurin acidic cluster sorting protein-2 (PACS-2) [117]. Calnexin has also been shown to regulate SERCA activity and control the availability of Ca^2+^ for mitochondrial respiration [118]. Another Ca^2+^-binding chaperone, calreticulin, determines the sensitivity of the ER cholesterol-sensing mechanism through the sterol regulatory-element binding protein (SREBP) pathway, which is crucial in maintaining membrane fluidity and ER stress levels [116]. Their implications in metabolic disease-related mechanistic links are encouraged for future research.

## Figures and Tables

**Table 1:** Key regulators of Ca^2+^ signaling in hepatic lipid metabolism.

Name	Subcellular Localization	Function	Involvement in NAFLD
InsP3R1	ER membrane	Ca^2+^ release from ER to cytoplasm	Increased expression is found in NASH patients; hepatic knockout mice are more resistant to lipid accumulation [46].
InsP3R2	ER membrane	Ca^2+^ release from ER to cytoplasm	InsP3R2 is down-regulated in NAFLD mouse models and NASH patients; null mice have no apparent lipid metabolic phenotype [51].
STIM1	ER membrane	Ca^2+^ sensor that drives Ca^2+^ entry from extracellular space	STIM1/2 inducible knockout mice have reduced lipolysis but increased lipophagy [95].
Orai	Plasma membrane	Ca^2+^ entry from extracellular space to cytoplasm	Orai1 is moderately increased in hepatic steatosis [34]; loss-of-function mutations impairs lipolysis but increases lipophagy [95].
SERCA2b	ER membrane	Ca^2+^ uptake from cytoplasm to ER	Impaired activity is associated with ER stress [36]; overexpression alleviates hepatic steatosis [37].
VDAC	Mitochondrial outer membrane	Entry of Ca^2+^ and other metabolites	
Mfn2	ER–mitochondria contacts	Forms dimer with Mfn1; ER–mitochondria tethering	Down-regulation is observed in NAFLD mouse models and NASH patients [59]; hepatic ablation results in ER stress and impaired insulin signaling [60].
MCU	Mitochondrial inner membrane	Ca^2+^ uptake to the mitochondria	Hepatic ablation of MCU delays cytoplasmic Ca2+ clearance and promotes lipid accumulation [71].
TPC2	Lysosome	Ca^2+^ release from the lysosome	TPC2-deficient mice are more susceptible to NAFLD when fed with a high-cholesterol diet [84].
Calreticulin	ER lumen	Ca^2+^ buffering; chaperone	Association with NAFLD is not determined; knockout cells have altered membrane fluidity and ER stress levels [116].
